# Effectiveness of Lumbar Drain Versus Hyperventilation to Facilitate Transsphenoidal Pituitary (Suprasellar) Adenoma Resection

**DOI:** 10.5812/aapm.6510

**Published:** 2013-03-26

**Authors:** Davood Aghamohamadi, Ali Ahmadvand, Firooz Salehpour, Rozita Jafari, Farid Panahi, Give Sharifi, Ali Meshkini, Abdolrasol Safaeian

**Affiliations:** 1 Department of Anesthesiology, School of Medicine, Imam Reza Teaching Hospital, Tabriz University of Medical Sciences, Tabriz, Iran; 2 Department of Neurosurgery, School of Medicine, Imam Reza Teaching Hospital, Tabriz University of Medical Sciences, Tabriz, Iran; 3 Department of Ear, Nose and Throat, School of Medicine, Imam Reza Teaching Hospital, Tabriz University of Medical Sciences, Tabriz, Iran; 4 Student Division of Research Development and Coordination Center (RDCC), School of Medicine, Tabriz University of Medical Sciences, Tabriz, Iran; 5 Neuroscience Research Center, School of Medicine, Tabriz University of Medical Sciences, Tabriz, Iran; 6 Department of Ear, Nose and Throat, School of Medicine, Shahid Beheshti University of Medical Sciences, Tehran, Iran; 7 School of Health and Nutrition, Tabriz University of Medical Sciences, Tabriz, Iran

**Keywords:** Hypercapnia, Lumbar Drain, Hyperventilation, Adenoma

## Abstract

**Background:**

Developing controlled hypercarbia is a known scheme of lowering the suprasellar part of the adenoma in order to assist the surgeon, which acts through raising the ICP and therefore the CSF pressure.

**Objectives:**

The purpose of this study is to compare the effect of introducing a lumbar drain with that of controlled hypercapnia on the quality of transsphenoidal pituitary tumor resection and CSF leak.

**Patients and Methods:**

Fifty two patients with pituitary adenoma who underwent transsphenoidal hypophysectomy by the same surgeon were included. They were randomly divided into two groups. A lumbar drain catheter introduced into the L3-L4 subarachnoid space under local anesthesia in all patients. The same anesthesia was performed in both groups. In the study group, we used a saline injection into the subarachnoid space versus hypoventilation in the control group in order to increase the ICP according to the surgeon's request. The surgeon's satisfaction during the tumor resection and the resection time were assessed during the surgery. The CSF catheter was closed and sent with the patient for CSF drainage. If there was no CSF leak, the catheter removed 24 hours later. With evidence of a CSF leak, we used the catheter as a lumbar drain. The time taken for the leakage control was assessed.

**Results:**

The satisfaction came from 21 (87.5%) and 2 (9.1%) for surgeon in the first and the second group respectively (P = 0.0001). CSF leakage time in the first and the second group was 1.6 ± 0.24 and 5 ± 0.50 respectively. It revealed a significant difference between the two groups (P = 0.001). The mean resection time was 13.54 ± 0.66 minutes in the study group; and 30.91 ± 0.98 minutes in the control group.

**Conclusions:**

In summary, the method described here for ICP manipulation is an effective procedure for a better visualization of the pituitary tumor during transphenoidal resection by surgeon and beneficial in managing the CSF leak following surgery.

## 1. Background

Transsphenoidal pituitary surgery has evolved over the past decades. The evolution is due to the technological advancements that have served as a catalyst for minimally invasive surgeries (1). Adenoma is usually approached transnasally. Traditionally, resection has been guided by the use of intraoperative fluoroscopy; however, computer-guided frameless stereoscopy may also be used (2). The endoscopic endonasal approach has become more common and may be associated with fewer complications as well as shorter recovery duration. In addition, the incidence of postoperative DI (Diabetes insipidus) may be less frequent in this approach (3). Transsphenoidal operations were originally performed on tumors within the sella turcica and they are also routinely performed on suprasellar tumors now. When a spontaneous prolapse of the tumor capsule or diaphragm does not occur, a descent may be facilitated by instilling 15 to 20 cc of air or lactated ringer's solution into a lumbar subarachnoid catheter. Alternatively, bilateral jugular vein compression or application of the end expiratory pressure can help in delivering a suprasellar tumor. In the case of a significant supracellar extension, a catheter may facilitate the descent of the tumor. As air is insufflated, video fluoroscopy can be used for better visualization of the suprasellar portion of the tumor. Similar results can be achieved with a valsalva maneuver or jugular vein compression (4). Following a successful resection of a tumor, a valsalva maneuver may be used to test for a CSF leak. If a CSF leak is readily observed, surgeons will mostly likely pack the sella with fat. The inhaled agents sevoflurane, desflurane, and isoflurane have all been shown to increase lumbar CSF pressure in normocapnic patients undergoing a transsphenoidal pituitary surgery. In the presence of raised ICP, nitrous oxide is usually avoided, and anaesthesia is maintained with intravenous agents. Hypocapnia should be avoided because a reduction of the brain bulk makes the access to any suprasellar extension more difficult (5).

## 2. Objectives

The aim of this study is to compare the effect of introducing a lumbar drain with that of controlled hypercapnia on the quality of pituitary tumor resection via transsphenoidal approach.

## 3. Patients and Methods

Fifty two patients with pituitary adenoma who underwent transsphenoidal hypophysectomy by the same surgeon were included. Imaging revealed suprasellar extension in all subjects. They were divided into two groups by a simple randomization. Inclusion criteria were an age range between 30-60 and the presence of a suprasellar macroadenoma. We excluded all patients with rising ICP, cardiovascular disease or COPD. In the operating room, the monitoring included an electrocardiogram, central vein pressure, and intracranial pressure, invasive and noninvasive blood pressure. There was no monitoring of CCP however it was calculated by (MAP-ICP = CPP) while saline injection and rising ICP therefore it was not monitored continuously. All of the patients were turned over to on to their sides. An 18 gauge catheter was introduced into the l3-l4 subarachnoid space under local anesthesia. A 50 cc syringe was attached to the CSF catheter via a three-way port for the injection of saline and monitoring of CSF pressure. The induction of anesthesia was conducted with Fentanyl 2μg/kg, Midazolam/01 mg/kg, Thiopentone 4-5 mg/kg, Cisatracuriom. /015 mg/kg; It was maintained with 50% N2O in 50% O2 and Isoflurane 1%-1.5% with an intermittent positive pressure ventilation to achieve an end-tidal CO2 around 30 mmHg. Further relaxation was maintained by repeating cisatracuriom. Following securing the airway with the endotracheal tube, the throat was packed to protect blood leakage into the stomach and causing postoperative nausea and vomiting. The patient was positioned with a 10° head-up tilt, and the surgery commenced. The baseline arterial blood gas was measured. By means of the technique assigned to the patient, the cerebrospinal fluid pressure was raised at the surgeon's request, usually when the floor of the sella was removed. The surgeon was unaware of the technique used. A screen was placed between the surgeon on one side and the anesthetists on the other side. In the first group, increments of 2.5 m of normal saline were purged via the spinal catheter up to 30 cc, and the descent of the tumor was assessed by the surgeon. At the same time ICP as well as CPP, was monitored and calculated. Then saline volume and the time to reach satisfactory exposure were recorded. In the second group, at first the circle absorber was switched off and the tidal volume and respiratory rate were decreased until end- Tidal (ET) CO2 reached to maximum level of CO2, 50mm Hg and again time to reach satisfactory was recorded. Complete visualization, descent of tumor, visualization of arachnoid or suprasellar capsule was considered satisfactory and was recorded. If the surgeon was satisfied prior achieving a CSF pressure of 20 mmHg or ETCO2 of 50 mm Hg, no further attempts were made, however if the pressure did not rise with our hypoventilation, we again used a saline infusion to raise the pressure. During the dissection, if an excessive descent of the central part of the tumor made the periphery not accessible, CSF pressure by CSF aspiration in the control group has been decreased or hyperventilated in the study group. The cerebral perfusion pressure was continuously monitored and kept above 60 mm Hg, and the mean blood pressure was maintained below 90 mmHg. Then the second arterial blood gas was measured. Muscle relaxation was reversed after the surgeon closed the sellar floor with subcutaneous fat. The patient was extubated and the CSF catheters were closed and sent with the patient for CSF drainage. If there was no CSF leak, the catheters were removed 24 hours later.

### 3.1. Statistical Analysis

The obtained data were expressed as Means standard deviations, frequencies and percentages. The data analyzed by an SPSS version 17. To compare the quantitative data, an independent sample T-test was used and for the qualitative data, a Chi-square test or Fisher's exact test was used. In all the investigated cases, the results were considered statistically significant in case of P ≤ 0.05.

## 4. Results

**Figure 1:**
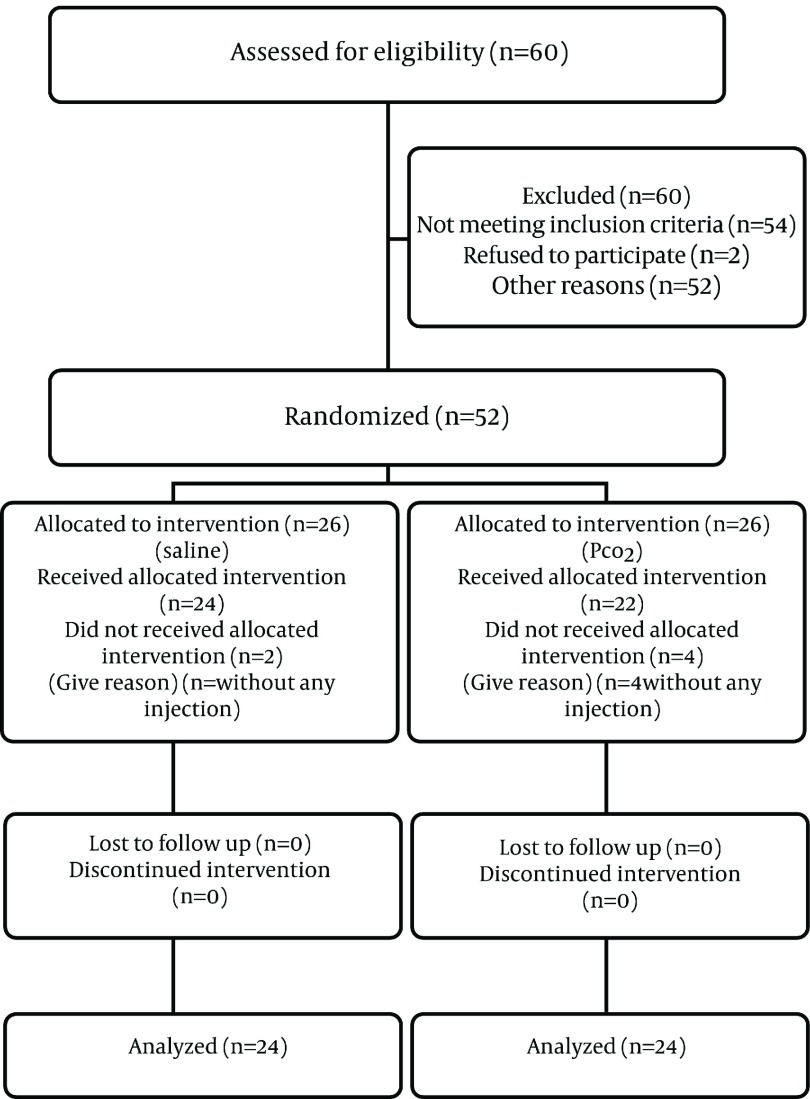
Statistical Analysis Algorithm of Patients

**Figure 2:**
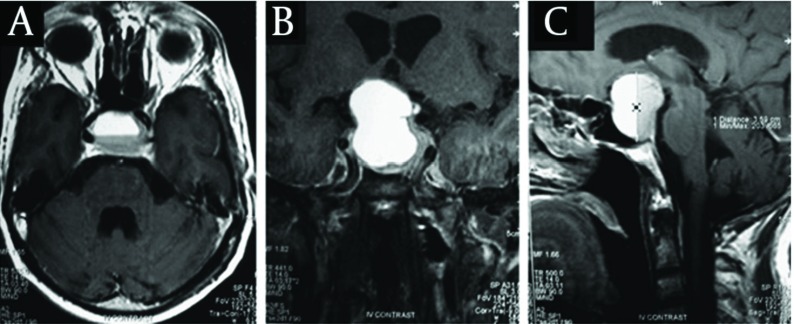
Preoperative Axial (A), Coronal (B), Sagittal (C) Magnetic Resonance Imaging Scan Following Contrast Injection of Patient Documenting a Suprasellar Pituitary Adenoma

Statistical analysis was used for 46 out of 52 patients (Figure 1). There were 12 males (50%) in the first group and 10 males (45.5%) in the second one. Twelve patients (50%) were female in the first group and the same number of females (54.5%) was in the second group. So, there was no significant difference between the two groups (P = 0.75). 0.91 ± 0.98 minutes in the first and the second groups. Therefore with P < 0.001 the difference was the mean age in the first group was (intrathecal slain injection) 45.5 ± 2.23 and in the second group (hypocapnea) was 45.32 ± 2.42. Also we obtained P = 0.948 which refers to no significant difference. The mean time of resection in the high ICP group was 13.54 ± 0.66 minutes, while it was significant. Regarding surgeon satisfaction in the first group, in 3 operations the surgeon (12.5%) was not satisfied however in 21 other operations (87.5%) he was satisfied. In the second group, in 20 operations (90.9%) the surgeon was not satisfied but in two operations (9.1%) he was. Regarding P = 0.0001, the difference was statistically significant. CSF leakage in the first and the second group was 1.6 ± 0.24 and 5 ± 0.50 days respectively, which showed a significant difference between the groups (P = 0.001). The preoperative axial, coronal and sagittal Magnetic Resonance Imaging scan following contrast injection of patient documenting a suprasellar pituitary adenoma are shown in Figure 2.

## 5. Discussion

Transsphenoidal microsurgery is a preferred approach for more than 95% of pituitary tumors and for a large proportion of other sellar abnormalities. Decompression of the intrasellar portion of the tumor frequently permits a suprasellar extension to prolapse into view within the sella. Following resection, the diaphragm prolapses and generally signifies that the resection is complete. When a spontaneous prolapse of the tumor capsule or the diaphragm does not occur, instillation of 15 to 20 cc of air or lactated Ringer’s solution into a lumbar subarchnoid catheter may facilitate the descent (4). Alternatively, bilateral jugular vein compression or application of positive end-expiratory pressure might be beneficial in delivering a suprasellar tumor. If the tumor still fails to descend, a ring curette can be used cautiously in the intracranial space. Following tumor removal and hemostasis has been achieved; the sella must be carefully evaluated with regard to reconstruction and closure (4). Spaziantean De Divitis described a pumping technique in which air is injected in increments of 10 cc to a maximum of 50 - 60 cc delivered to the subarachnoid space in this way; a complete visualization of the tumor was made possible (6). Nowadays, intrathecal normal saline or ringer is used with ICP monitoring to outline the tumors (4). In the study of Korula they revealed the operating condition in the same manner .Surgeons in 20 operations in the study group while in 17 operations of the control group. No unusual side effects occurred while using both techniques. They found that hypercarbia was satisfactory in raising CSF pressure up to 20 mmHg, and the surgical descent of the suprasellar portion of the pituitary tumor was as effective as with intrathecal saline (6). However it has yet to be determined how long should commence resection following injection. In addition, hypercapnia may run the risk of bleeding with rising blood pressure. Additional studies are required to validate this method (5). However limiting PaCO2 to 60 mmHg minimizes the deleterious side effects of hormocapnia (40-45 mm Hg) hypertension, tachycardia and increased myocardial load. Therefore the preferred target is High-N in our study, the duration between raising ICP and resection in the hypercapnic and saline groups were 30.91 and 13.54 minutes respectively. Various methods have been introduced to decrease the rate of postoperative CSF leak, but the incidence remains the same. A wide range of CSF leakage rates have been reported. Some authors have already reported an incidence that ranges from 2 to 33% (7). Magliulo and his associate reported a 17.6% CSF leak, while Nutick and Korol reported a 13% incidence. Fishman, as well as Ruiz - Fornells and colleagues, reported a 17% and a 15% CSF leakage respectively (4, 8). On the other hand, there is a minimal risk associated with the use of CSF lumbar drains (9). Alexander et al. reported a 23% reduction in CSF leakage by means of CSF lumbar drains. They reported no neurologic complication (8). Prophylactic CSF drainage may decrease the postoperative CSF leakage rate by numerous factors. It can reduce the effect of a transient postoperative hydrocephalus and also improve surgical exposure by decompressing the posterior fossa, which allows less cerebellar retraction to be used, thereby decreasing the rebound swelling of the of cerebellum in the posterior fossa (4, 8). It can decrease the pressure gradient to zero across the fistula (10). Following a transsphenoidal surgery (TSS), the routine insertion of an elective lumbar drainage in patients in whom intaoperative CSF leakage is observed significantly reduces the incidence of postoperative meningitis (11). The adverse events commonly observed with lumbar spinal fluid drainage are infections, neurological injury caused by spinal drain insertion, pneumocephalus, haematomas, and CSF overdrainage (12). Robert found 2.5% headaches in 530 patients; there was no evidence of neurologic dysfunction (6). On the other hand, there are some report about the brain stem and cerebellar dysfunctions (13). In the present study, CSF leak happened in 27% and 19% of the patients in the hypercapnea and the saline group respectively. The duration for an effective treatment of CSF leak was 5 ± 0.5 and 1.6 ± 0.24. It revealed the efficacy of introducing a lumbar drain for ICP manipulation before surgery and how it preserves assistance to manage CSF leak subsequently. In summary, the method described here for ICP manipulation is an effective procedure for a better visualization of the pituitary tumor during transphenoidal resection by the surgeon and is helpful in managing CSF leak following surgery.
